# Beneficial effects of natural flavonoids on neuroinflammation

**DOI:** 10.3389/fimmu.2022.1006434

**Published:** 2022-10-24

**Authors:** Yu Chen, Fu Peng, Ziwei Xing, Junren Chen, Cheng Peng, Dan Li

**Affiliations:** ^1^State Key Laboratory of Southwestern Chinese Medicine Resources, School of Pharmacy, Chengdu University of Traditional Chinese Medicine, Chengdu, China; ^2^Department of Pharmacology, Key Laboratory of Drug-Targeting and Drug Delivery System of the Education Ministry, Sichuan Engineering Laboratory for Plant-Sourced Drug and Sichuan Research Center for Drug Precision Industrial Technology, West China School of Pharmacy, Sichuan University, Chengdu, China

**Keywords:** natural flavonoids, neuroinflammation, microglia, astrocytes, brain disorders

## Abstract

Neuroinflammation is the fundamental immune response against multiple factors in the central nervous system and is characterized by the production of inflammatory mediators, activated microglia and astrocytes, and the recruitment of innate and adaptive immune cells to inflammatory sites, that contributes to the pathological process of related brain diseases, such as Alzheimer’s disease, Parkinson’s disease, depression, and stroke. Flavonoids, as a species of important natural compounds, have been widely revealed to alleviate neuroinflammation by inhibiting the production of pro-inflammatory mediators, elevating the secretion of anti-inflammatory factors, and modulating the polarization of microglia and astrocyte, mainly *via* suppressing the activation of NLRP3 inflammasome, as well as NF-κB, MAPK, and JAK/STAT pathways, promoting Nrf2, AMPK, BDNF/CREB, Wnt/β-Catenin, PI3k/Akt signals and SIRT1-mediated HMGB1 deacetylation. This review will provide the latest and comprehensive knowledge on the therapeutic benefits and mechanisms of natural flavonoids in neuroinflammation, and the natural flavonoids might be developed into food supplements or lead compounds for neuroinflammation-associated brain disorders.

## Introduction

Neuroinflammation generally refers to a complex immune response in the central nervous system (CNS) to various endogenous or exogenous stimuli, such as misfolded proteins, toxins and pathogen, leading to brain tissue inflammatory cell infiltration, gliosis, neuronal loss, etc. (1). Pro-inflammatory mediators, produced by microglia, astrocytes and other immune cells in the process of neuroinflammation, repress the differentiation and trigger apoptosis and necroptosis of neurons, increase the production of excitatory neurotransmitters and inhibit the transmission of monoamine neurotransmitter, ultimately resulting in neuronal degeneration (2–4). And amyloid-β (Aβ), tau, α-synuclein and other misfolded proteins aggregate in neurons or intercellular neurons during inflammation, that forms neurofibrillary tangles and senile plaques in cerebral cortex and hippocampus, and generates Lewy bodies in substantia nigra pars compacta (5, 6). Furthermore, the integrity of tight junctions of endothelial cells and the components of basal lamina are degraded in inflammatory states, which augments the permeability of blood-brain barrier (BBB) and subsequently leads leukocytes invading the brain parenchyma (7, 8). Therefore, intervening neuroinflammation may be an important strategy to the treatment of brain disorders.

## The physiological and pathological mechanisms of neuroinflammation

The roles of neuroinflammation are different, normally, inflammation is a defense mechanism that initially protects the brain *via* clearing up pathogens, cell fragments, mis-folded proteins and other stimulus to maintain or restore the integrity of tissues (9). Nevertheless, uncontrolled neuroinflammation engenders neuronal degeneration and BBB disruption, that is marked by the secretion of pro-inflammatory cytokines, chemokines, and small-molecule messengers, which are primarily released by activated microglia and astrocytes (10).

Microglia are macrophages derived from erythromyeloid pro-genitors in the yolk sac, and exhibit a wide array of functions that include regulation of programmed cell death of neurons, striping excess synapses from developing neurons and promotion of neurite formation (11). With the change of brain microenvironment, microglia, like peripheral macrophages, are activated by various inflammatory stimuli, and polarize into classical M1 type and alternative M2 type (12). Specific as follows, M1 microglia are typically characterized by the secretion of pro-inflammatory cytokines and chemokines, such as IL-6, IL-1β, TNF-α, and MCP-1, bringing about unbridled and prolonged neuroinflammation (13). On the contrary, M2 microglia with the markers of Ym-1, FIZZ-1 and Arg-1, secrete anti-inflammatory cytokines, including IL-4, IL-10, and IL-13, to suppress inflammation (14).

Astrocytes are neural parenchymal cells derived from neural stem cells, and are able to regulate the extracellular balance of ions, fluid and transmitters, modulate cerebral blood flow and the formation and maintenance of the BBB (15). In the process of inflammation, astrocytes polarize into neurotoxic phenotype (A1), that is characterized by cellular hypertrophy, increased production of glial fibrillary acidic protein and complement, astrogliosis, and glial scars formation, in addition to pro-inflammatory factors secretion (16). Besides, A1 astrocytes directly influence vascular and perivascular cells leading to alterations in BBB permeability (17). In contrast, astrocytes are activated by protective factors in inflammation and polarize into neuroprotective phenotype (A2), that increase the release of neurotrophic factors, such as BDNF, NGF and VEGF, as well as thrombospondins like TSP-1, which promote outgrowth and survival of neurons (18, 19).

Cellular crosstalk among microglia, astrocytes and neurons poses feedback loops and brings maladjusted and self-magnifying neuroinflammation. Normally, astrocytes offer nutritional support molecules for microglia to promote their morphological and functional stability, while in the process of neuroinflammation, A1 astrocytes release inflammatory mediators and increase the permeability of BBB to activate M1 microglia accompanied by an enhanced ability to migrate (20, 21). Meanwhile, molecular factors secreted by M1 microglia also polarize astrocytes into A1 state inducing astrocytosis and the secretion of neurotoxic factors (22). Furthermore, proinflammatory mediators released by A1/M1 directly initiate neuronal apoptosis and necroptosis, and due to the decrease in A1/M1 uptake capacity, intercellular excitatory transmitters such as glutamate are increased, resulting in neuronal excitotoxicity (23, 24). Conversely, A2/M2 secrete anti-inflammatory cytokines, neurotrophic factors and other protective mediators to inhibit neuroinflammation and promote neuronal generation and survival (25). Therefore, the functional changes of microglia and astrocytes affect neuronal function and central nervous immune system, and bring about the occurrence or aggravation of various brain diseases.

Overall, an increase of inflammatory mediators, polarization of microglia and astrocytes, and crosstalk among microglia, astrocytes and neurons, are the key factors for the occurrence and development of neuroinflammation, thereby, adjusting the above changes are effective strategies to treat neuroinflammation-related brain disorders.

## Effects of natural flavonoids on neuroinflammation

Flavonoids, natural compounds with a basic structural unit of 2-phenylchromone, are widely present in herbs and various dietary sources, such as fruits, vegetables, tea, and cereal in the form of glycosides or free state. Human interventions and experimental studies have shown a role of natural flavonoids in brain diseases, as evidenced by the reduction in multiple pro-inflammatory mediators. Therefore, understanding the effects and mechanisms of flavonoids in anti-neuroinflammation would be highly valuable for developing nutritional guidelines and therapeutic strategies to related brain disorders.

### Effects of flavones and flavonols on neuroinflammation

**Figure 1:**
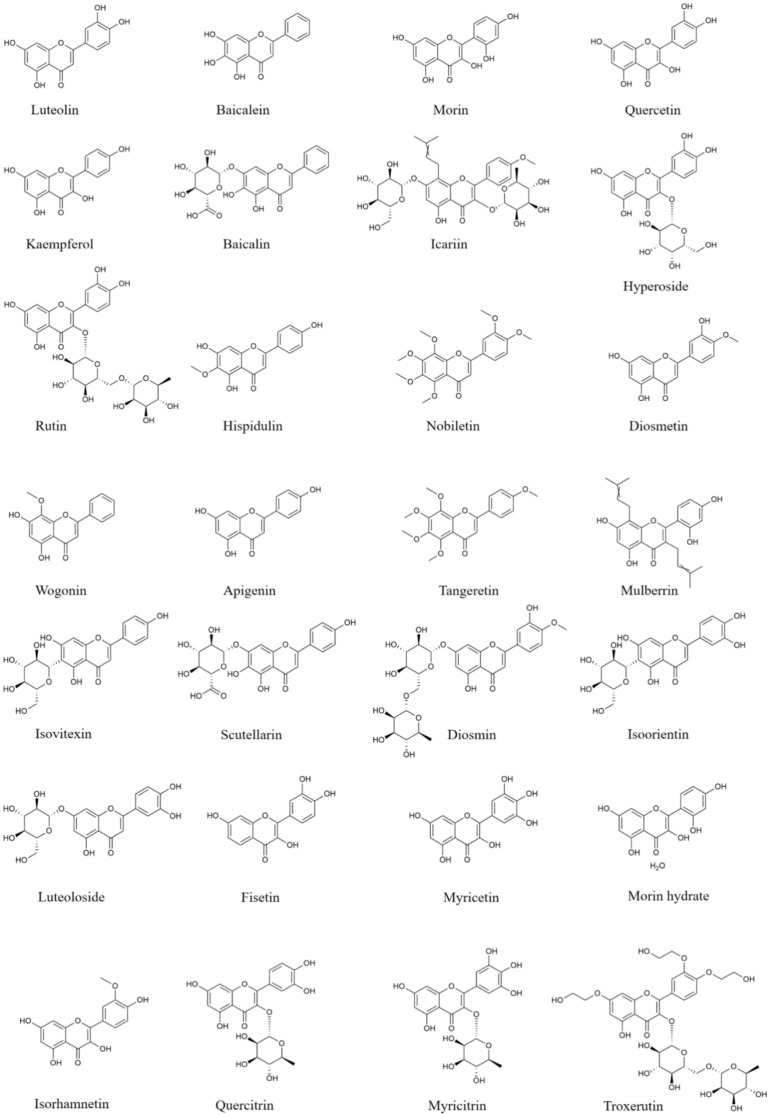
Structures of flavones and flavonols with anti-neuroinflammatory effects.

Flavones and flavonols are important groups of flavonoids that are widely studied. Luteolin (**Figure 1**), a flavone compound, is found in various herbs, vegetables, and fruits, such as perilla leaf, peppermint, celery, carrot, and apple. Luteolin is reported to down-regulate the secretion of IL-1β, TNF-α, and IL-6 (26–28), suppress p65 and p38 phosphorylation in lipopolysaccharide (LPS)-induced C6 cells (26), inhibit nucleus p65, ASC, NLRP3, and cleaved-Caspase-1 protein expression, and increase Nrf2 protein in oxyhemoglobin-induced primary cortical neurons and glia cells (27, 28). In LPS, Aβ_1-42_ and triple-transgenic-induced AD mice, luteolin ameliorates behavior impairment, inhibits overproduction of pro-inflammatory mediators (26, 29–31), as well as restrains GFAP, p38 protein expression and the phosphorylation of JNK and p65 (26, 31). In other studies, luteolin is reported to decrease the release of pro-inflammatory mediators (27, 28), decrease TRAF6, TLR4, and p-p65 expression as well as TRAF6 ubiquitination in the brain of intracerebral hemorrhage (ICH)-induced rats (27), suppress NLRP3 proteins expression and increase the activity of Nrf2 in the brain of subarachnoid hemorrhage-induced rats (28). Therefore, luteolin might inhibit the excessive production of pro-inflammatory cytokines through regulating TLR4/TRAF6/NF-κB, MAPK, Nrf2 pathways and NLRP3 inflammasome to treat neuroinflammation-related brain diseases.

Baicalin (**Figure 1**) and baicalein (**Figure 1**) are rich in *Scutellaria baicalensis* Georgi, an edible medicinal plant. Various disease models, such as neurodegenerative diseases and encephalomyelitis have proved the anti-neuroinflammatory effects of baicalin and baicalein. In ischemia-reperfusion (I/R) and chronic unpredictable mild stress (CUMS)-induced mice, baicalin supplementation leads a reduction of IL-6, IL-1β, and TNF-α (32, 33), a trend of decreased TLR4 protein expression, and an increase of phosphorylation of PI3k, Akt and FoxO1 in the hippocampus of mice (33). Moreover, baicalin decreases IL-18 and iNOS levels (34–37), suppresses the protein expression of Iba-1, GFAP, TLR4, p-p65, p-IκBα, NLRP3, and cleaved-Caspase-1 in the hippocampus of APP/PS1 mice (37), reduces the production of HMGB1 and NF-κB, and elevates SIRT1 expression in the cerebral cortices and hippocampus from LPS-induced mice (34–36). Thus, baicalin possesses the ability to attenuate neuroinflammation *via* adjusting NLRP3 inflammasome and PI3k/Akt/FoxO1, SIRT1/HMGB1, and TLR4/NF-κB signaling pathways.

Baicalein, an aglycone of baicalin, is also widely studied in neuroinflammation. Baicalein inhibits microglia activation and polarization with decreasing TNF-α, iNOS, IL-1β, IL-6, CD16 and CD86 production, and enhancing Arg-1 and CD206 levels in LPS plus IFN-γ-induced BV2 cells through activating STAT1 expression and inhibiting TLR4/NF-κB pathway (38), and in ischemic penumbra from middle cerebral artery occlusion (MCAO)-induced rats through the inactivation of IκBα, JNK, ERK and p38, as well as nuclear translocation of p65 (38–40). In other studies, baicalein is reported to reduce IFN-γ, IL-5, and IL-12 secretion, as well as repress GFAP and Iba-1 expression in substantia nigra (SN) and midbrain from MPTP or rotenone-induced PD mice *via* downregulating cleaved-Caspase-1, cleaved-GSDMD, and NLRP3, as well as promoting PSD95, SYP, BDNF, p-TrkB, CREB, p-PI3k, p-Akt, and p-CaMK II expression (41, 42). In summary, baicalein restrain microglia activation and polarization through inhibiting NLRP3 inflammasome and regulating MAPKs, STAT1, TLR4/NF-κB, BDNF/TrkB/CREB signaling pathways.

Morin, 3,5,7,2’,4’-pentahydroxyflavone, is a bioactive flavonol compound that is extensively found in a variety of herbs, vegetables and fruits, like onion, orange, mulberries and almond hulls. Lots of researches have intensely demonstrated the anti-neuroinflammatory properties of morin (43, 44). Morin has been reported to decrease the secretion of NO, TNF-α, and IL-6, and suppress the protein expression of NF-κB in the striatum, prefrontal cortex and hippocampus from social defeat stress-induced mice (45) and in the hippocampus from Aβ_1-42_-induced AD rats (46). Besides, morin represses the production of nNOS and GFAP in brain of ifosfamide-induced neurotoxicity rats *via* decreasing the production of NF-κB and JNK, and increasing Nrf2 expression (47). These suggest that morin could suppress neuroinflammation *via* JNK, NF-κB and Nrf2 signaling pathways.

Quercetin (**Figure 1**), a natural flavonol is widely distributed in herbal medicines, fruits and vegetables, such as tea, apple, grape, and onion. In LPS-induced primary microglia or BV2 cells, quercetin decreases the ability of phagocytic, reduces the levels of inflammatory mediators including NO, TNF-α, IL-6, IL-1β, MCP-1, CXCL10, iNOS, COX-2, and lipocalin-2, and increases the secretion of IL-10 through activating AMPK and Nrf2 signaling pathways, as well as inhibiting NF-κB signaling pathway (48–50). *In vivo* studies, quercetin is reported to improve aging-, or LPS-induced behavior disorders, inhibit microglia and astrocytes activation, as well as decrease IL-1β levels *via* elevating SIRT1 protein expression and suppressing NLRP3, cleaved-Caspase-1 protein production in the brain of mice (49–51). Besides, in LPS-stimulated or traumatic brain injury rats, quercetin decreases the production of pro-inflammatory mediators in rat brain through suppressing NF-κB pathway, as well as initiating the Nrf2/HO-1 pathway (52). Thus, quercetin is a safe and effective dietary supplement to ameliorate neuroinflammation *via* increasing SIRT1 protein expression, inhibiting NLRP3 inflammasomes activation and adjusting NF-κB, Akt, AMPK, and Nrf2/HO-1 pathways.

Kaempferol (**Figure 1**), a dietary flavonol, presents in most plant-based foods, such as tea, broccoli, kale, cabbage and grapefruit, which has been described to possess resultful anti-neuroinflammatory effects. In LPS-induced BV2 cells, kaempferol reduces iNOS, IL-1β, IL-18, and TNF-α levels, suppresses CD32 production, and enhances Arg-1 and CD206 expression through down-regulating NLRP3, ASC, Caspase-1, p-p38 and p-NF-κB (53, 54). *In vivo* studies, kaempferol diminishes the production of COX-2, MCP-1, ICAM-1, IL-1β, IL-6, and TNF-α, and attenuates microglia activation in striatum of LPS-induced mice *via* suppressing the protein expression of HMGB1 and TLR4 (55), and in ischemic cortices from I/R rats through decreasing the phosphorylation and nuclear transposition of p65 (56). Kaempferol also decreases iNOS, COX-2 and IL-18 production through inhibiting NF-κB, p38 phosphorylation and NLRP3 inflammasome activation in the SN from PD rats induced by 6-hydroxydopamine (6-OHDA) (53). These researches suggest that kaempferol could regulate microglia polarization and reduce the pro-inflammatory mediators *via* suppressing NLRP3, HMGB1/TLR4, MAPKs, and NF-κB signaling pathways.

Icariin (**Figure 1**), a typical flavonol glycoside isolated from *Epimedium brevicornu* Maxim. has been studied to treat a variety of inflammation-related brain disorders. In LPS-treated glia, icariin down-regulates NO, TNF-α, IL-1β, IL-18, COX-2, and iNOS levels (57, 58). In MPTP- or 6-OHDA-induced PD mice, icariin alleviates dopaminergic neuronal damage, decreases the secretion of pro-inflammation cytokines and inhibits the protein expression of Iba-1 and GFAP in brain through suppressing NLRP3 inflammasome activation and promoting Nrf2, Keap1, HO-1 and NQO1 protein production (59, 60). In other study, icariin also reduces the levels of pro-inflammatory mediators including IFN-γ, MCP-1, IL-12, IL-17A, and GM-CSF in serum and brain of APP/PS1 mice (61). Thus, icariin shows great potentiality to attenuate neuroinflammation.

A natural flavonol glycoside, hyperoside (**Figure 1**) isolates from many herbs, such as *Cuscuta chinensis* Lam., *Forsythia suspensa*, and *Crataegus pinnatifida* Bge. Numerous studies have pointed out that hyperoside possesses anti-neuroinflammatory effects. Hyperoside supplement is reported to alleviate IL-1β, IL-6, IL-8, and TNF-α secretion through up-regulating SIRT1, Wnt1, β-Caspase, Shh, and Patch in LPS-treated HT22 cells (62). And in MPTP-induced PD mice, hyperoside reverses the motor dysfunction, reduces pro-inflammatory factors production and down-regulates Iba-1 and GFAP *via* reducing NLRP3, ASC, and P20 expression and increasing PACAP content and CREB phosphorylation in the SN (63). Furthermore, in streptozotocin plus high-fat diet-induced type 2 diabetic neuropathy rats, hyperoside alleviates cognitive dysfunction, and decrease the production of IL-1β, IL-6, TNF-α, and iNOS through suppressing NF-κB and Caspase-3 proteins expression in the brain (64). Therefore, hyperoside has great potential to alleviate neuroinflammation *via* inhibiting NLRP3 inflammasome activation, as well as regulating SIRT1/Wnt and NF-κB pathways.

Rutin (**Figure 1**), a flavonol glycoside abundantly distributed in tea, buckwheat, passion flower, and apple, exerts potent anti-neuroinflammatory properties. In LPS-treated BV2 cells, rutin promotes the phenotypic transformation of M1 to M2 with reducing IL-6, TNF-α, IL-1β, NO, iNOS, and CD86 levels, and up-regulating Arg-1, CD206 and IL-10 *via* inhibiting the expression of TLR4 and MyD88, and blocking NF-κB and IKKβ phosphorylation (65). Rutin is also found to improve Tau-P301S-induced memory deficits, suppress the activation of microglia and astrocytes, as well as decrease the levels of pro-inflammatory mediators, through the inactivation of IKKβ and p65 in the brain of AD mice (66). Thus, rutin shows great potential to ameliorate neuroinflammation *via* TLR4/MyD88/NF-κB signaling pathway.

Moreover, other flavones and flavonols compounds, such as hispidulin, cymaroside, myricitrin and troxerutin also exert anti-neuroinflammatory effects. They could restrain the activation and polarization of microglia, as well as inhibit the expression of pro-inflammatory mediators *via* suppressing NLRP3 inflammasome activation and regulating PI3k/Akt, MAPKs, Nrf2 or NF-κB signaling pathways in PD, AD, traumatic brain injury, depression and I/R injury, which are specifically showed in **Table 1**.

**Table 1:** Effects of flavones and flavonols on neuroinflammation.

Compound	Model	Type of disease/disorder	Index	Pathway	Ref
Hispidulin	LPS-induced BV2 cells	Neuroinflammation	↓: pro-inflammatory mediators	inhibit Akt/STAT3/NF-κB pathway	(67)
Isovitexin	LPS-induced BV2 and mouse primary cortical microglia cells, LPS-induced mice	Neuroinflammation	↓: pro-inflammatory mediators ↑: M2 polarization	promote CaMKKβ/AMPK-PGC-1α signaling pathway	(68)
Scutellarin	LPS-induced primary astrocytes, LPS-induced mice, LPS-induced rats	Neuroinflammation, depression	↓: pro-inflammatory mediators, GFAP, Iba-1 ↑: IL-4	inhibit TLR4/NF-κB pathway and NLRP3 inflammasome	(69, 70)
Nobiletin	LPS-induced BV2 cells, LPS-induced mice and rats	Neuroinflammation, depression	↓: pro-inflammatory mediators, Iba-1 ↑: IL-10	promote AMPK pathway, inhibit MAPKs, Akt, NF-κB pathways and NLRP3 inflammasome	(71, 72)
	APP/PS1 mice	AD	↓: pro-inflammatory mediators, GFAP	inhibit NLRP3 inflammasome	(73)
Isoorientin	APP/PS1 mice	AD	↓: pro-inflammatory mediators, Iba-1	inhibit NF-κB pathway	(74)
Diosmin	rotenone-induced rats	PD	↓: pro-inflammatory mediators	inhibit NF-κB pathway	(75)
Mulberrin	LPS-induced BV2 cells, MPTP-induced rats	PD	↓: pro-inflammatory mediators, Iba-1, GFAP	promote Wnt/β-catenin pathway	(76)
Diosmetin	*Streptococcus pneumoniae*-induced bacterial meningitis in rats	Bacterial meningitis	↓: pro-inflammatory mediators	inhibit PI3k/Akt/NF-κB pathway	(77)
Wogonin	Kainate-induced temporal lobe epilepsy in rat	Epilepsy	↓: pro-inflammatory mediators	inhibit NF-κB pathway	(78)
Apigenin	Acrylonitrile-induced neurotoxicity in rats	Neurotoxicity	↓: pro-inflammatory mediators, Caspase-3, Caspase-9, Bax ↑: Bcl-2	inhibit HMGB-1/TLR4/NF-κB pathway	(79)
Luteoloside	MCAO-induced rats	Ischemic stroke	↓: pro-inflammatory mediators	regulate PPARγ/Nrf2/NF-κB pathway	(80)
Tangeretin	MACO-induced rats	Ischemic stroke	↓: pro-inflammatory mediators ↑: anti-inflammatory mediators	inhibit TLR4/NF-κB pathway	(81)
Troxerutin	I/R-induced rats	Ischemic stroke	↓: pro-inflammatory mediators ↑: anti-inflammatory mediators	inhibit NLRP3 inflammasome	(82)
	LPS-induced rats	Neuroinflammation	↓: pro-inflammatory mediators	promote SIRT1/SIRT3 pathway and inhibit NF-κB pathway	(83)
Myricetin	LPS-induced BV2 cells, LPS-induced mice	Neuroinflammation	↓: pro-inflammatory mediators, Iba-1	inhibit MAPKs pathway	(84)
Myricitrin	LPS-stimulated mice	Neuroinflammation	↓: pro-inflammatory mediators	inhibit MAPKs and TLR4/HMGB1/NF-κB pathways	(85)
	Cecal ligation and puncture-induced rats	Sepsis-associated encephalopathy	↓: pro-inflammatory mediators, NLRP3	inhibit NF-κB and NLRP3 pathways	(86)
Fisetin	Cecal ligation and puncture-induced sepsis-associated encephalopathy in rats	Sepsis-associated encephalopathy	↓: pro-inflammatory mediators	inhibit NF-κB pathway	(87)
Morin hydrate	Chronic unpredictable stress-induced mice	Memory impairment	↓: pro-inflammatory mediators	inhibit NF-κB pathway	(88)
Quercitrin	LPS-induced mice	Depressive	↓: pro-inflammatory mediators	inhibit PI3k/Akt/NF-κB pathway, promote CREB/BDNF pathway	(89)
Isorhamnetin	High-fat and high fructose diet-induced mice	Metabolic syndrome-related cognitive complications	↓: pro-inflammatory mediators, MMP-1, MMP-3, MMP-9, Iba-1	inhibit NF-κB and MAPKs pathways	(90)

“↑” indicates promotion, “↓” indicates inhibition.

### Effects of flavanones and flavanonols on neuroinflammation

Pinocembrin is a kind of flavanone mainly extracted from honey and propolis. Pinocembrin inhibits microglia activation, reverses the up-regulation of TNF-α, iNOS, COX-2, IL-6, and IL-1β in the hippocampus from intermittent hypoxia-induced mice *via* suppressing the protein expression of NLRP3, ASC, and cleaved-Caspase-1, as well as enhancing BNIP3, LC3-II, ATG7, Beclin-1, and ATG5 expression (91). In other study, pinocembrin is found to alleviate CUMS-induced depressive-like behaviors, reduce pro-inflammatory cytokines and increase IL-10, TGF-β secretion in the hippocampus through the up-regulation of Nrf2 and HO-1, and the inactivation of NF-κB (92). Thus, pinocembrin has great potential to alleviate neuroinflammation through regulating NLRP3 inflammasome activation, BNIP3-mediated mitophagy, Nrf2/HO-1 along with NF-κB signaling pathways.

**Figure 2:**
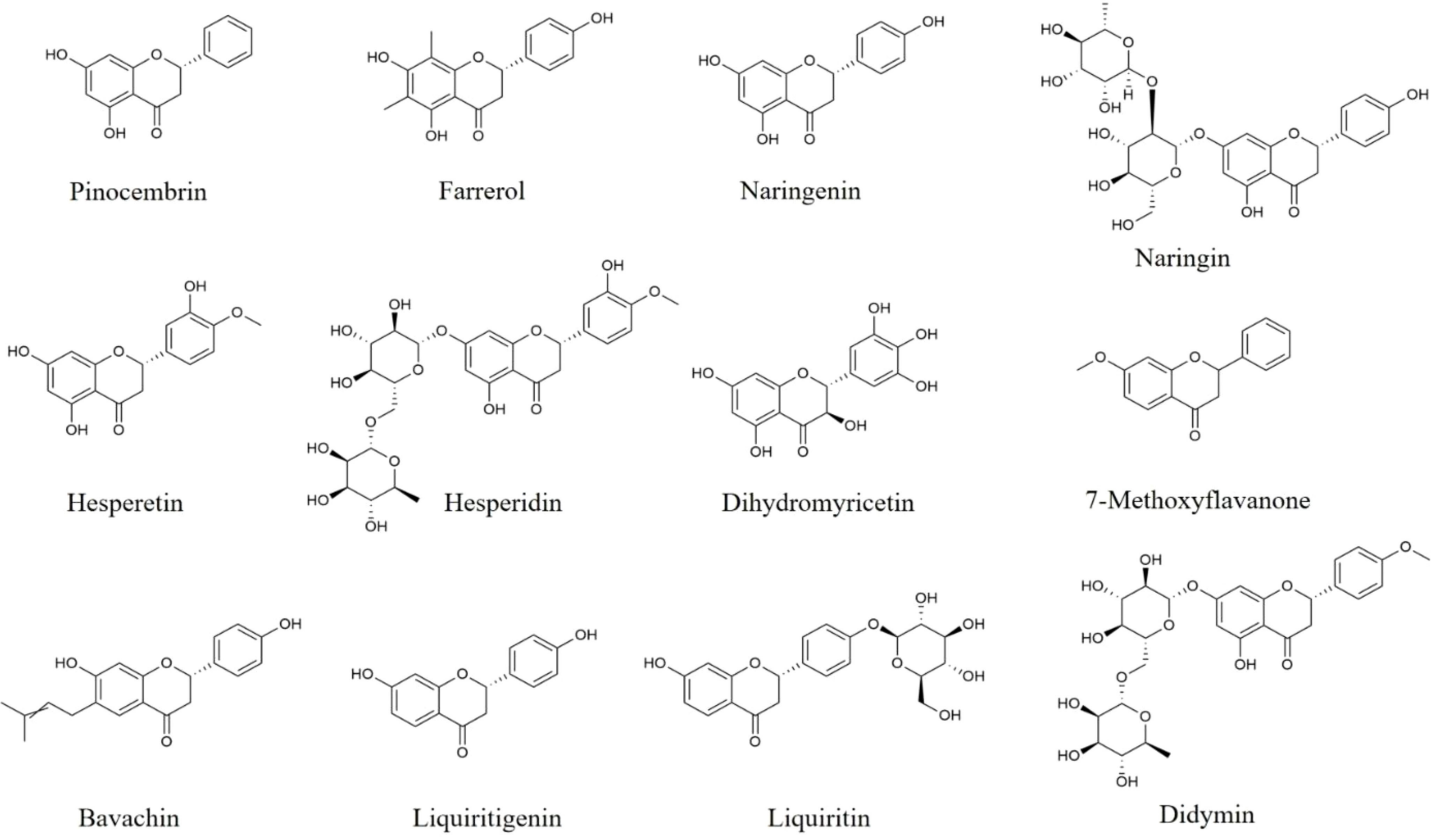
Structures of flavanones and flavanonols with anti-neuroinflammatory effects.

Farrerol (**Figure 2**), a type of 2,3-dihydroflavonoid, isolated from rhododendron leaves, down-regulates the expression of IL‐6, IL‐1β, TNF‐α, iNOS, COX‐2, NO, and PGE2 in LPS-induced BV2 cells through inhibiting p65 and Akt phosphorylation (93). And in MPP^+^‐treated BV2 cells, farrerol is also found to decrease pro-inflammatory mediators levels *via* suppressing TLR4 and MyD88 expression, as well as p65 and IκBα phosphorylation (94). Moreover, farrerol alleviates motor dysfunction and mitigates microglial activation in the SN of LPS-induced rats (93). Thus, farrerol exerts anti-neuroinflammatory effects through regulating Akt and TLR4/MyD88/NF-κB pathways.

Naringin and its aglycone naringenin (**Figure 2**) are widely found in citrus fruits as a biological neuroactive flavanones compound which has anti-neuroinflammatory activities. In social-defeat stress-induced neurobehavioral deficits mice and MCP-1-stimulated rats, naringin is reported to reverse behavioral impairments, and reduce TNF-α, IL−1β, IL-6, and NO secretion in striatum, prefrontal cortex, and hippocampus (95, 96). Moreover, naringin could also cut pro-inflammatory mediators down in the brain of haloperidol−revulsive or bisphenol-A-mediated rats (97, 98). Besides, in LPS-induced BV2 cells, naringenin, the aglycone of naringin, is found to inhibit pro-inflammatory factors such as NO, IL-1β, and IL-18 release, and up-regulate Arg-1, and IL-10 through suppressing NLRP3 and cleaved-Caspase-1 protein expression, and inhibiting JNK and ERK phosphorylation (99, 100). In other study, naringenin improves the cognitive deficiency, decreases pro-inflammatory cytokines secretion, and inhibits GFAP protein expression in the hippocampus from AD mice (101). These results suggest that naringin and naringenin play beneficial roles in neuroinflammation and related diseases.

Hesperidin and its aglycone hesperetin (**Figure 2**) with anti-neuroinflammatory effects, are mainly distributed in citrus fruits such as oranges, grapefruit, and lemons. Hesperidin is found to inhibit the release of TNF-α, IL-1β, IL-6, as well as MCP-1 in LPS-stimulated BV2 cells and HT22 cells and the brain of N-methyl-D-aspartate-induced mice through inhibiting NF-κB pathway (102). Besides, hesperidin also down-regulates the levels of pro-inflammatory mediators, decreases HMGB1, RAGE, p-IκBα, and p-p65 protein expression, and increases the protein levels of BDNF and p-TRκB in corticosterone-stimulated PC12 cells and hippocampus of CUMS-processed mice (103).

In LPS-stimulated BV2 cells, hesperetin, an aglycone of hesperidin, inhibits the levels of NO, and iNOS *via* the inactivation of TLR4, ERK, p38, and p65 (104, 105). And in LPS-induced mice and Aβ_1-42_-induced AD mice, hesperetin is found to improve behavioral disorders, suppress astrocyte and microglia activation, and decrease iNOS and COX-2 production in the cortical and hippocampus of mice *via* inhibiting the protein expression of TLR4 and p-p65 (104–106). On the basis of their safety and effectiveness, hesperidin and hesperetin may be further researched to alleviate neuroinflammation as food supplements.

Dihydromyricetin (**Figure 2**) is a major bioactive flavanonol extracted from *Ampelopsis grossedentata*. In LPS-stimulated BV2 cells, dihydromyricetin reduces the levels of TNF-α, IL-6, IL-1β, COX-2, and iNOS through inhibiting NLRP3, ASC, Caspase-1, HIF1a, TLR4 and MyD88 protein expression, as well as Akt, p65, and IκBα phosphorylation (107, 108). Besides, in the brain from LPS-induced mice, dihydromyricetin is reported to down-regulate pro-inflammatory mediators, and suppress CD11b and CD14 expression through TLR4/Akt/HIF1a/NLRP3 pathway (107). Dihydromyricetin supplement reduces the secretion of IFN-γ, IL-1α, MIP-1β, CXCL2, CCL17, IL-2, and IL-7 in serum, and improves the loss and dystrophy of microglia in the hippocampus from social isolation-induced mice (109). Moreover, dihydromyricetin also ameliorates the memory deficiency and reduces the levels of pro-inflammatory mediators in the brain of Aβ_1-42_-processed AD rats (110). These results suggest that dihydromyricetin could alleviate neuroinflammation-related brain disorders.

In addition, other flavanones and flavanonols compounds, such as 7-Methoxyflavanone, dihydroquercetin, liquiritigenin exert anti-neuroinflammatory effects which are amply shown in **Table 2**.

**Table 2:** Effects of flavanones, flavanonols, isoflavones, chalcones, dihydrochalcone and other flavonoids on neuroinflammation.

Category	Compound	Model	Type of disease/disorder	Index	Pathway	Ref
Flavanones and flavanonols	7-Methoxyflavanone	LPS-induced BV2 cells, LPS-induced mice	Neuroinflammation	↓:pro-inflammatory mediators, Iba-1	inhibit TLR4/MyD88/MAPKs pathway and activate Nrf2/NQO-1 pathway	(111)
	Bavachin	LPS-induced BV2 cells or primary microglial cells	Neuroinflammation	↓: pro-inflammatory mediators ↑: anti-inflammatory mediators, M2 polarization	inhibit TRAF6/NF-κB pathway and NLRP3 inflammasome	(112)
	Liquiritin	LPS-treated mice	Depression	↓: pro-inflammatory mediators, Iba-1	enhance FGF-2 expression	(113)
	Liquiritigenin	Aβ-treated N2a or BV2 cells, APP/PS1 mice	AD	↓: pro-inflammatory mediators, Iba-1, M1 polarization ↑: M2 polarization	inhibit NLRP3 inflammasome	(114)
	Didymin	ICH-induced mice	ICH	↓: pro-inflammatory mediators, MPO, Iba-1	inhibit NLRP3 inflammasome	(115)
Isoflavones	Isoformononetin	STZ-induced neuroinflammation in rats	Neuroinflammation	↓: pro-inflammatory mediators, GFAP, Iba-1	inhibit NLRP3 inflammasome and NF-κB pathway	(116)
	Biochanin A	LPS-stimulated BV2 cells	Neuroinflammation	↓: pro-inflammatory mediators, ROS	inhibit TLR4/MyD88/NF-κB, PI3k/Akt and ERK pathways	(117)
	Ononin	Aluminium chloride-provoked AD rats	AD	↓: pro-inflammatory mediators	inhibit NF-κB and MAPKs pathways, increase BDNF and PPAR-γ	(118)
Chalcones and dihydrochalcones	Butein	LPS-induced co-culture of BV2 cells and SH-SY5Y cells	Neuroinflammation	↓: pro-inflammatory mediators ↑: cell viability	inhibit NF-κB and MAPKs pathways	(119)
	Isobavachalcone	LPS-induced BV2 cells, LPS-induced mice	Neuroinflammation	↓: pro-inflammatory mediators	inhibit TRAF6/NF-κB pathway and activate Nrf2/HO-1 pathway	(120)
	Xanthohumol	APP/PS1 mice	AD	↓: pro-inflammatory mediators	activate mTOR/LC3 pathway	(121)
		LPS-induced mice	Depression	↓: pro-inflammatory mediators, ROS, Iba-1, GFAP	inhibit NF-κB pathway and activate Nrf2/HO-1 pathway	(122)
	Isoliquiritin	LPS plus ATP-induced primary microglia cells, LPS-induced mice and chronic social defeat stress-induced mice	Depression	↓: pro-inflammatory mediators	inhibit NF-κB pathway and NLRP3 inflammasome	(123)
Anthocyanidins	Cyanidin-3-O-Glucoside	LPS-stimulated BV2 cells	Neuroinflammation	↓: pro-inflammatory mediators	inhibit NF-κB and MAPKs pathways	(124)
Biflavonoid	Agathisflavone	LPS- or IL-1β-induced co-culture of neuron and glial	Neuroinflammation	↓: pro-inflammatory mediators, M1 polarization, Iba-1, GFAP, ↑: M2 polarization	inhibit NF-κB pathway	(125)
	Isoginkgetin	LPS-induced BV2 cells, LPS-induced depression in mice	Neuroinflammation, depression.	↓: pro-inflammatory mediators, ROS, Iba-1	inhibit NF-κB and MAPKs pathways	(126)
	Ginkgetin	MACO-induced ischemic stroke in rats	Ischemic stroke	↓: pro-inflammatory mediators	inhibit TLR4/NF-κB, and JAK2/STAT3 pathways	(127)

“↑” indicates promotion, “↓” indicates inhibition.

### Effects of isoflavones on neuroinflammation

**Figure 3:**
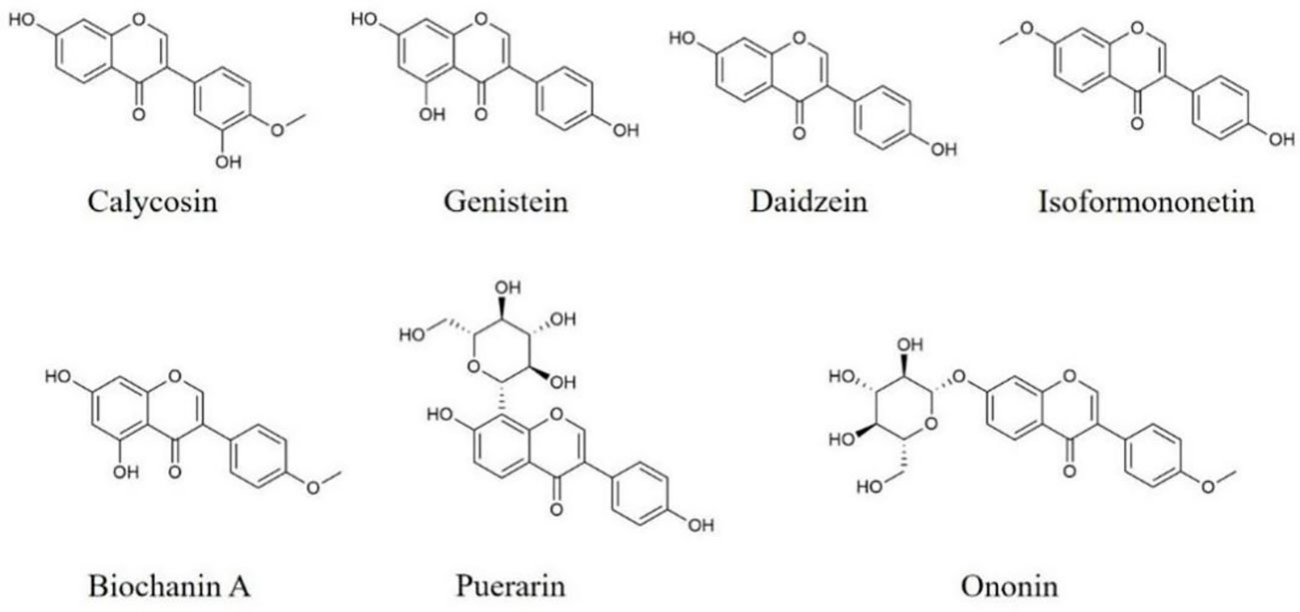
Structures of isoflavones with anti-neuroinflammatory effects.

Calycosin (**Figure 3**) is an active isoflavone isolated from Radix Astragali. Treatment of calycosin protects mice against ICH-induced damages, improves neurobehavior, reduces the secretion of TNF-α, IL-6, IL-1β and IL-18, as well as inhibits microglia activation in perihematomal tissue of the brain from ICH-induced mice through suppressing IκBα and p65 phosphorylation, and repressing both transcriptional and translational of NLRP3, ASC, Caspase-1 (128). Besides, calycosin mitigates the behavioral dysfunctions, protects TH neurons and down-regulates the levels of pro-inflammatory mediators in the brain from MPTP‐induced PD mice *via* suppressing TLR2, TLR4, and nuclear NF-κB expression, as well as inhibiting p38, JNK and ERK phosphorylation (129). Thus, calycosin can alleviate neuroinflammation through modulating NLRP3 inflammasome and MAPKs, TLR/NF-κB pathways.

Genistein (**Figure 3**) distributed in soy is an isoflavone and has anti-neuroinflammatory effects. Genistein ameliorates hypoxic-ischemic brain damage-induced neuroinflammation with reducing TNF-α, IL-1β, and IL-6 secretion by the up-regulation of Nrf2, HO-1 and IκBα, and the inactivation of IκBα and NF-κB (130). Moreover, genistein improves cognitive disorders, and reduces MCP-1 release, and elevates the levels of IL-10, IGF-1, BDNF, and CREB in the hippocampus of hypoxia-induced mice (131). In other study, genistein is reported to reduce the levels of IL-6, IL‐1β, TNF-α, IL-8, iNOS, and CD16, and increase CD206 and Arg-1 expression in isoflurane-mediated BV2 cells and hippocampal regions from isoflurane-induced rats through restraining TLR4, MyD88, and TRAF6 protein expression, as well as suppressing TAK1, p38, ERK, IκBα and NF-κB phosphorylation (132). These evidences demonstrate that genistein is able to repress neuroinflammation *via* promoting Nrf2 pathway, as well as suppressing MAPKs and NF-κB pathways.

Puerarin (**Figure 3**), distributed in *Pueraria lobata*, could reduce the secretion of IL-8, IL-18, MCP-1 and CCL2 in TNF-α plus IL-1β-induced primary nerve cells and the trigeminal ganglions from complete Freund’s adjuvant-treated mice through the inhibition of NLRP3, Caspase-1, TGF-β1, NLRP1 protein and Smad3 phosphorylation, and the up-regulation of SIRT1 (133). Another study shows that puerarin could alleviate ICH-induced behavioral defects, drop hematoma volume and histological injury, decrease IL-6, IL‐1β, and TNF-α secretion, as well as down-regulate 3-NT, 8-OHdG, and ROS levels in the perihematomal brain tissue of ICH-induced rats *via* promoting PI3k and Akt phosphorylation, and suppressing the phosphorylation of p65 and the nuclear accumulation of p65 (134). Thus, puerarin inhibits the activation of NLRP3 inflammasome, promotes the expression of SIRT1 and regulates TGF-β1/Smads, PI3k/Akt, and NF-κB pathways to ameliorate neuroinflammation.

In addition, daidzein, isoformononetin, ononin and other isoflavonoids compounds are also able to inhibit pro-inflammatory cytokines secretion *via* regulating Akt, ERK and NF-κB signaling pathways to exert anti-neuroinflammatory effects which are shown in **Table 2**.

### Effects of chalcones and dihydrochalcone on neuroinflammation

**Figure 4:**
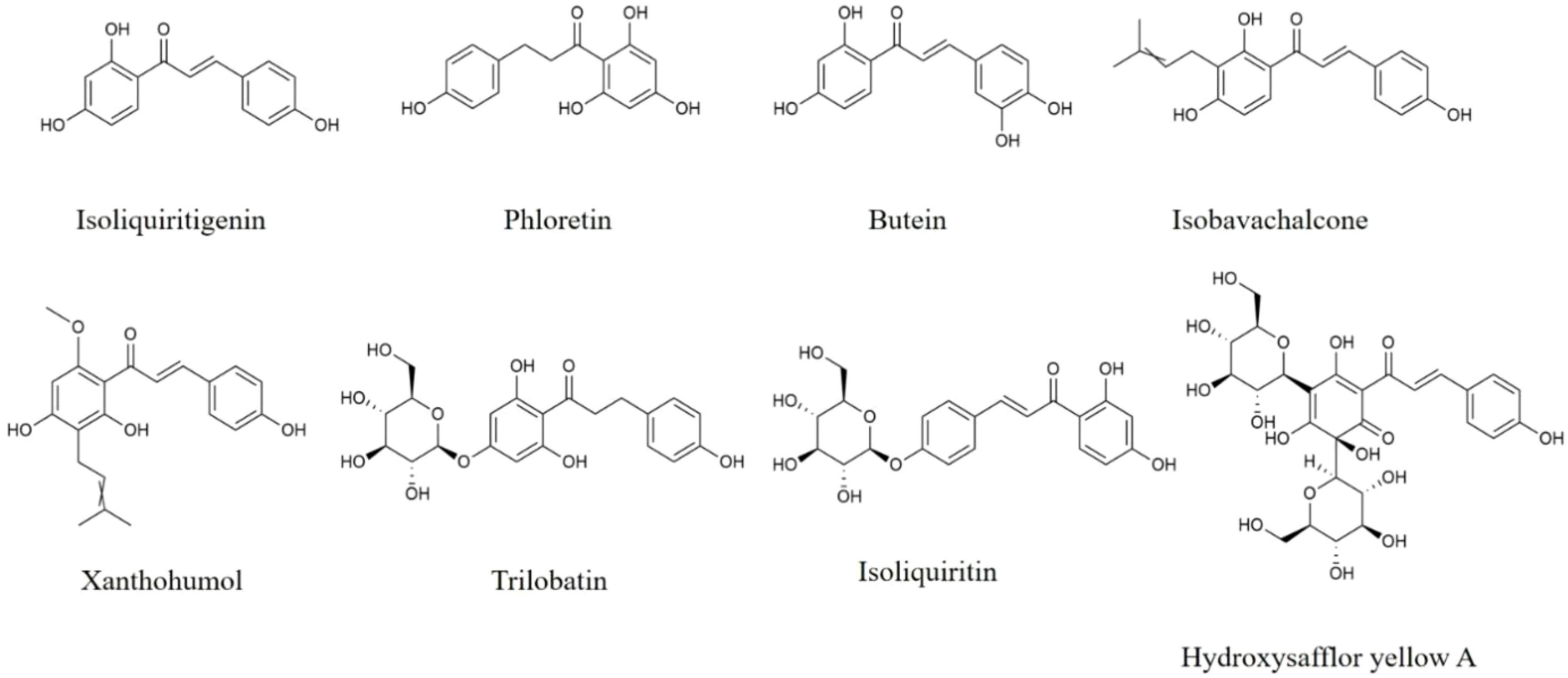
Structures of chalcones and dihydrochalcone with anti-neuroinflammatory effects.

Isoliquiritigenin (**Figure 4**), with a chalcone structure, is derived from licorice root. Isoliquiritigenin alleviates morphological changes and reduces the levels of COX-2, iNOS, NO, IL-1β, IL-6, and TNF-α *via* the up-regulation of Nrf2, HO-1 and NQO1, and the inhibition of p65 nuclear translocation in BV2 cells induced by Aβ oligomers, and increases the cell viability in co-culture of BV2 cells and N2a cells (135). *In vivo* study, isoliquiritigenin reverses LPS-induced cognitive deficits, promotes the expression of PSD-95, BDNF, and synaptophysin, and restrains the secretion of CCL3, TNF-α, IL-1β, and IL-6 in hippocampus from LPS-stimulated rats *via* increasing GSK-3β phosphorylation and Nrf2, HO-1, NQO1 expression, as well as suppressing the protein expression of NF-κB (136). Moreover, isoliquiritigenin is also reported to improve cognitive impairment, decrease TNF-α, IL-1β and IL-18 secretion, and suppress the activation of microglia and astrocytes in hippocampus from kainic acid-induced seizures rats through inhibiting cleaved-Caspase-3, cleaved-Caspase-9, and NLRP3 expression, and enhancing Nrf2, HO-1, and NQO1 production (137). Therefore, isoliquiritigenin shows great potential to attenuate neuroinflammation by regulating NLRP3 inflammasome, Nrf2/HO-1 and NF-κB pathways.

Hydroxysafflor yellow A (HSYA) (**Figure 4**) exists in *Carthamus tinctorius* L. with good effects of alleviating neuroinflammation. HSYA treatment inhibits NO, TNF-α, IL-1β, IL-6, and iNOS levels, suppresses CD16 and CD32 expression, as well as promotes Arg-1 and CD206 production in LPS-induced primary microglia through the up-regulation of Nrf2, HO-1 and SIRT1 (138). HSYA also reduces pro-inflammatory cytokines release, increases the secretion of IL-4, IL-10 and IL-13, as well as regulates microglia polarization in Aβ_1-42_-stimulated BV2 cells *via* suppressing the expression of TREM2, and TLR4, and the phosphorylation of p65 and IκBα (139). Administration of HSYA down-regulates the levels of iNOS and COX-2 in 6-OHDA-revulsive SH-SY5Y cells and SN from 6-OHDA-induced mice through suppressing IκBα production and the phosphorylation of p65, p38 and JNK (140). Furthermore, HSYA also lessens the cerebral infarction area, decreases the levels of NO and iNOS, and increases the production of eNOS in the cortical penumbra of I/R rats *via* elevating the expression of IκBα and p-GSK-3β, and suppressing cleaved-Capase-3 expression and p65 phosphorylation (141). Thus, HSYA could suppress the activation and polarization of microglia, and reduce pro-inflammatory mediators production through TREM2/TLR4/NF-κB, Nrf2/HO-1, and MAPK pathways.

Trilobatin (**Figure 4**) is a dihydrochalcone compound distributed in *Lithocarpuspolystachyus* Rehd., and has anti-neuroinflammatory effects. Trilobatin is reported to improve cognitive impairment, reduce activated microglia and astrocytes with decreasing Iba-1 and GFAP expression, and inhibit the secretion of TNF-α, IL-1β and IL-6 in the hippocampus from APP/PS1 and triple-transgenic-induced AD mice through the down-regulation of HMGB1, TLR4, MyD88, TRAF6 and p-p65 (142, 143). Moreover, in OGD/R-stimulated astrocytes and the brain of MACO-induced I/R rats, trilobatin reduces the production of iNOS, and suppresses the activation of microglia and astrocytes *via* up-regulating Nrf2, HO-1, NQO1 and SIRT3 protein, as well as suppressing p65 phosphorylation and the expression of Keap1, TLR4, MyD88, and TRAF6 (144). Thus, as a potential therapeutic drug, trilobatin can prevent and treat neuroinflammation-related brain disorders.

Phloretin (**Figure 4**), a dihydrochalcone flavonoid, is abundant in apple with anti-neuroinflammatory effects. Phloretin is reported to down-regulate the secretion of TNF-α in the brain of Aβ_25-35_-induced AD rats (145), decrease the levels of IL-6, IL-1β, iNOS and COX-2, and reduce activated microglia and astrocytes in the brain from MPTP-induced PD mice (146).

In addition to the above compounds, the other chalcones and dihydrochalcone compounds such as phloridzin, xanthohumol, isoliquiritin also have anti-neuroinflammatory effects *via* modulating mTOR, NF-κB, or Nrf2/HO-1 pathways, which are shown in **Table 2**.

### Effects of others flavonoids on neuroinflammation

**Figure 5:**
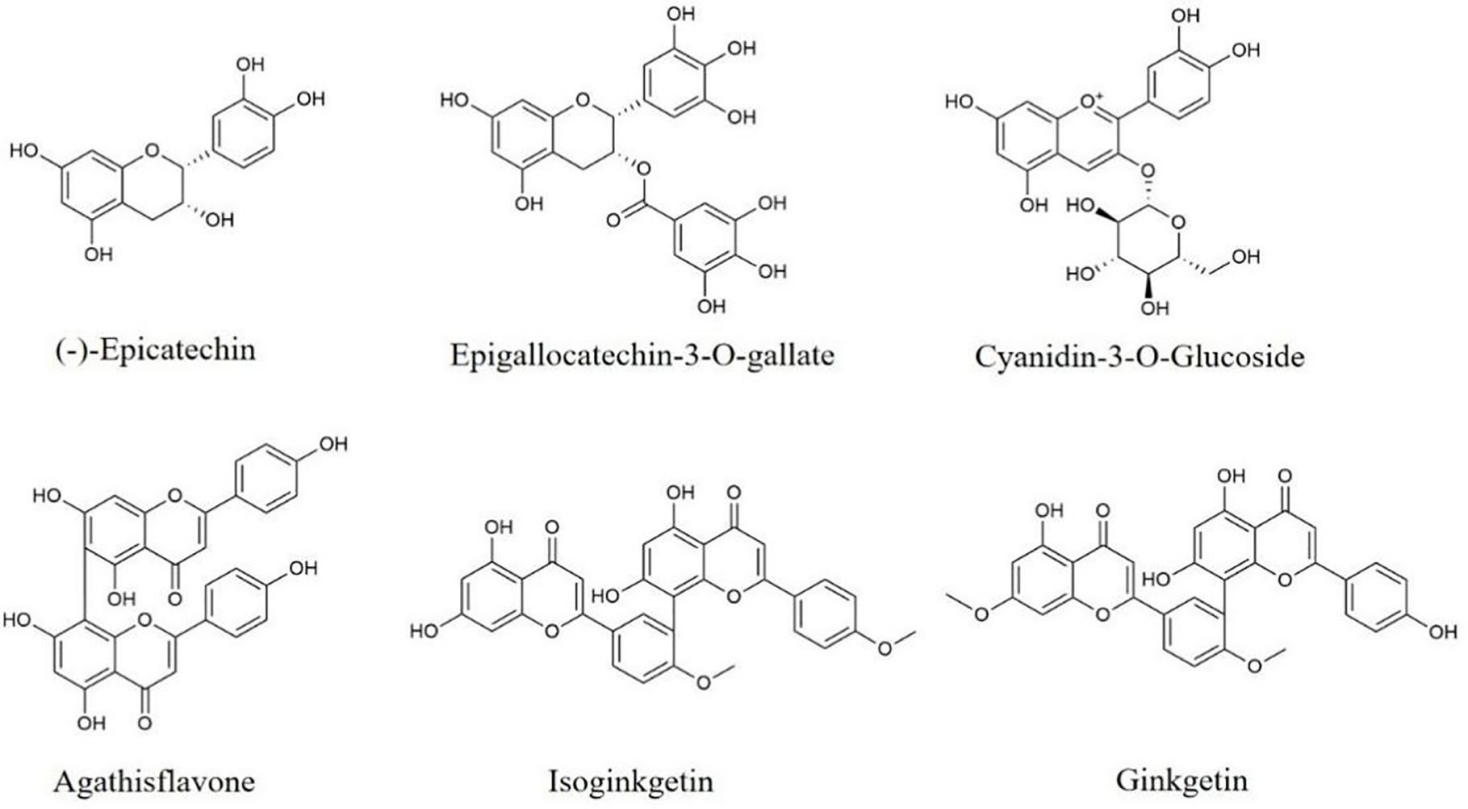
Structures of other flavonoids with anti-neuroinflammatory effects.

There are others types of flavonoids, including flavanols, anthocyanidins, and bioflavonoids. Some of them also have anti-neuroinflammatory effects, such as flavanol epigallocatechin-3-O-gallate (EGCG), anthocyanidin cyanidin-3-O-glucocide, bioflavonoid isoginkgetin, etc.

(-)-Epicatechin (**Figure 5**), a dietary flavanols, is widely distributed in foods such as tea, cocoa and grapes. Treatment of (-)-Epicatechin improves HFD-induced cognitive and memory impairment and inhibits the activation of microglia *via* decreasing the transcription of TLR4 and NOX4 in the hippocampus (147). In other study, (-)-Epicatechin represses the activation of microglia and astrocytes, reduces the levels of TNF-α, IFN-γ, IL-1β, IL-3, IL-5, IL-6, IL-15, and COX-2, as well as promotes IL-10 and IL-11 secretion in the hippocampus from aging mice through suppressing Caspase-3, Caspase-9 and NF-κB protein expression, and promoting Akt and GSK-3β phosphorylation (148). Therefore, (-)-Epicatechin possesses ability to lighten neuroinflammation through regulating TLR4/NOX4, Akt/GSK-3β, and NF-κB pathways.

EGCG (**Figure 5**) with anti-neuroinflammation effects is the major catechin component from green tea. EGCG supplementation down-regulates the levels of TNF-α, IL-6, and IL-1β in palmitic acid-stimulated BV2 cells and hypothalamus of HFD mice through suppressing the expression of JAK2 and STAT3 (149). In APP/PS1 transgenic mice, EGCG is reported to improve behavioral disorders, inhibit microglia activation, decrease IL-1β secretion and increase the release of IL-10 and IL-13 in the hippocampus (150). EGCG also down-regulates NO and TNF-α levels, as well as inhibits Caspase-3 and NF-κB protein expression in the cerebral cortex and hippocampus from ethanol-treated rats (151). Besides, in CUMS-induced rats and rotenone-stimulated PD rats, EGCG also ameliorates behavioral disorders, and reduces pro-inflammatory cytokines secretion (152, 153). Thus, EGCG, as a dietary compound, treats neuroinflammation-related brain diseases by modulating JAK2/STAT3 and NF-κB pathway.

Moreover, in PD, depression or ischemic stroke models, the other flavonoids compounds such as cyanidin-3-O-glucocide, agathisflavone and ginkgetin also exert anti-neuroinflammatory effects *via* PI3k/Akt, TLR4/NF-κB and MAPK pathways, which are shown in **Table 2**.

## Conclusion and perspective

**Figure 6:**
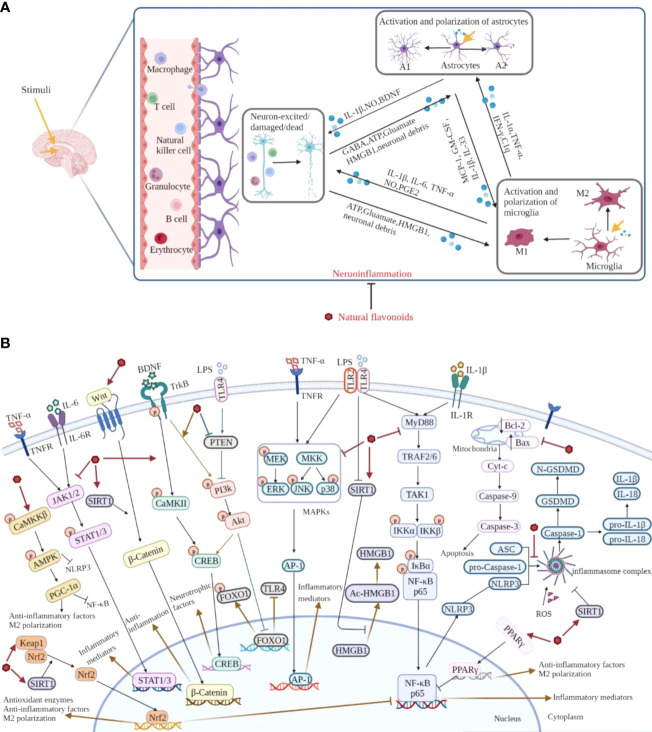
Overview of the pathological course of neuroinflammation and the mechanisms of natural flavonoids against neuroinflammation. **(A)** Under neuroinflammation state, microglia and astrocytes are activated and secrete a lot of inflammatory cytokines, chemokines, along with small-molecule messengers which disturb the normal functioning of neurons and cause damage in brain tissue. **(B)** Natural flavonoids play anti-neuroinflammatory effects *via* inhibiting the activation of NLRP3 inflammasome and MAPKs, JAK/STAT, NF-κB pathways, promoting Nrf2, AMPK, BDNF/CREB, Wnt/β-Catenin, PI3k/Akt pathways and SIRT1-mediated HMGB1 deacetylation.

Neuroinflammation, a complex immune response, is a key hallmark of brain disorders. Following stimuli, activated microglia and astrocytes secrete massive pro-inflammatory cytokines, chemokines and small-molecule messengers, and cause further tissue dysfunction, which is the characteristic of neuroinflammation. Evidently, it is potential to develop therapeutic and preventive strategies to treat brain disorders targeting neuroinflammation. Based on the recent investigations, natural flavonoids exhibit plenty of beneficial anti-neuroinflammatory effects, such as down-regulating the expression of pro-inflammatory mediators, accelerating the secretion of anti-inflammatory cytokines, inhibiting astrocytosis, and suppressing the activation and polarization of microglia, and the main mechanisms of natural flavonoids against neuroinflammation include the inhibition of NLRP3 inflammasome activation and MAPKs, JAK/STAT, NF-κB and apoptotic pathways, as well as the activation of Nrf2, AMPK, BDNF/CREB, Wnt/β-Catenin, PI3k/Akt pathways and SIRT1-mediated HMGB1 deacetylation (**Figure 6**). And from the summary of current research, different types of natural flavonoids share with similar anti-neuroinflammatory mechanisms without obvious difference, among which, inflammatory and oxidative signaling pathways have been widely studied, while other pathways are less studied and need further study. Moreover, the current researches on natural flavonoids against neuroinflammation have some limitations. Firstly, the current researches mainly focus on flavones and flavonols, other types of flavonoids are less studied. Therefore, this is an area that warrants further investigation on rest of the flavonoids in related brain diseases. Secondly, the mechanism researches pay close attention to the activation and polarization of microglia, but ignore astrocytes and the crosstalk among microglia, astrocytes and neurons which requires further study. Overall, these preclinical data help us to further investigate natural flavonoids and offer ideas for finding new dietary supplements or lead compounds to treat neuroinflammation and related brain disorders.

## Author contributions

YC and FP contributed equally to the design and draft of the manuscript. YC and FP drafted the manuscript. YC, FP, ZX, and JC revised the manuscript. CP and DL conceived and designed the whole project. All authors reviewed and approved the final manuscript.

## Funding

Financial support by National Natural Science Foundation of China (82104477, U19A2010 and 81891012), National Interdisciplinary Innovation Team of Traditional Chinese Medicine (ZYYCXTD-D-202209) are gratefully acknowledged.

## Conflict of interest

The authors declare that the research was conducted in the absence of any commercial or financial relationships that could be construed as a potential conflict of interest.

## Publisher’s note

All claims expressed in this article are solely those of the authors and do not necessarily represent those of their affiliated organizations, or those of the publisher, the editors and the reviewers. Any product that may be evaluated in this article, or claim that may be made by its manufacturer, is not guaranteed or endorsed by the publisher.
